# Deciphering Interactions in Moving Animal Groups

**DOI:** 10.1371/journal.pcbi.1002678

**Published:** 2012-09-13

**Authors:** Jacques Gautrais, Francesco Ginelli, Richard Fournier, Stéphane Blanco, Marc Soria, Hugues Chaté, Guy Theraulaz

**Affiliations:** 1 Centre de Recherches sur la Cognition Animale, UMR-CNRS 5169, Université Paul Sabatier, Toulouse, France; 2 CNRS, Centre de Recherches sur la Cognition Animale, Toulouse, France; 3 Service de Physique de l'État Condensé, CEA-Saclay, Gif-sur-Yvette, France; 4 Istituto dei Sistemi Complessi, Consiglio Nazionale delle Ricerche, Roma, Italy; 5 Institute for Complex Systems and Mathematical Biology, King's College, University of Aberdeen, Aberdeen, United Kingdom; 6 Laboratoire Plasma et Conversion d'Energie, UMR-CNRS 5213, Université Paul Sabatier, Toulouse, France; 7 CNRS, Laboratoire Plasma et Conversion d'Energie, Toulouse, France; 8 Institut de Recherche pour le Développement (IRD), UMR EME, La Réunion, France; Princeton University, United States of America

## Abstract

Collective motion phenomena in large groups of social organisms have long fascinated the observer, especially in cases, such as bird flocks or fish schools, where large-scale highly coordinated actions emerge in the absence of obvious leaders. However, the mechanisms involved in this self-organized behavior are still poorly understood, because the individual-level interactions underlying them remain elusive. Here, we demonstrate the power of a bottom-up methodology to build models for animal group motion from data gathered at the individual scale. Using video tracks of fish shoal in a tank, we show how a careful, incremental analysis at the local scale allows for the determination of the stimulus/response function governing an individual's moving decisions. We find in particular that both positional and orientational effects are present, act upon the fish turning speed, and depend on the swimming speed, yielding a novel schooling model whose parameters are all estimated from data. Our approach also leads to identify a density-dependent effect that results in a behavioral change for the largest groups considered. This suggests that, in confined environment, the behavioral state of fish and their reaction patterns change with group size. We debate the applicability, beyond the particular case studied here, of this novel framework for deciphering interactions in moving animal groups.

## Author Summary

Swarms of insects, schools of fish and flocks of birds display an impressive variety of collective patterns that emerge from local interactions among group members. These puzzling phenomena raise a variety of questions about the behavioral rules that govern the coordination of individuals' motions and the emergence of large-scale patterns. While numerous models have been proposed, there is still a strong need for detailed experimental studies to foster the biological understanding of such collective motion. Here, we use data recorded on fish barred flagtails moving in groups of increasing sizes in a water tank to demonstrate the power of an incremental methodology for building a fish behavior model completely based on interactions with the physical environment and neighboring fish. In contrast to previous works, our model revealed an implicit balancing of neighbors position and orientation on the turning speed of fish, an unexpected transition between shoaling and schooling induced by a change in the swimming speed, and a group-size effect which results in a decrease of social interactions among fish as density increases. An important feature of this model lies in its ability to allow a large palette of adaptive patterns with a great economy of means.

## Introduction

Collective motion occurs across a variety of scales in nature, offering a wealth of fascinating phenomena which have attracted a lot of attention [1]–[5]. The self-organized motion of social animals is particularly intriguing because the behavioral rules the individuals actually follow and from which these remarkable collective phenomena emerge often remain largely unknown due to the tremendous difficulties to collect quality field data and/or perform controlled experiments in the laboratory. This situation does not prevent a thriving modeling activity, thanks to the relative ease by which numerical simulations can be conducted. However, most models of moving animal groups are built from general considerations, educated guesses following qualitative observations, or ideas developed along purely theoretical lines of thought [6]–[9]. Even when authors strive to build a model from data, as in the recent paper by Lukeman et al. [10], this model building amounts to writing down a fairly complicated structure a priori, involving many implicit assumptions, and to fit collective data to determine effective parameters, yielding a best-fit model.

On the other hand, recent studies within the physics community of simple, minimal models for collective motion have revealed an emerging picture of universality classes [11]–[15]: Take, for instance, the Vicsek model, arguably one of the simplest models exhibiting collective motion. In this model, point particles move at constant speed and choose, at discrete time-steps, their new heading to be the average of that of their neighbors located within unit distance. Many of these behavioral restrictions can be relaxed without changing the emerging collective properties. Fluctuations of speed can be allowed, some short-range repulsion (conferring a finite size to the particles) can be added, even explicit alignment can be replaced by inelastic collisions, etc., all these changes will still produce the remarkable nonlinear high-density high-order bands emerging near onset of collective motion, and, deeper in the ordered moving phase, the anomalously strong number fluctuations which have become a landmark of the collective motion of polarly aligning self-propelled particles [16]–[20]. The Vicsek model, in this context, is one of the simplest members of a large universality class defined by all models sharing the same large-scale properties. This universality class can be embodied in the continuous field equations that physicists are now able to derive. With such a viewpoint, different models in this class merely differ in the numerical values of their parameters [21]–[23], very much like different fluids are commonly described by the Navier-Stokes equations and differ only in their viscosity and other constitutive parameters.

Significant features nevertheless may be altered when a qualitatively important feature is changed, such as the symmetry of the aligning interaction, or added, as when local attraction/repulsion between individuals is also considered [8], [24] In this latter case, for instance, no strong clustering and high density band appears when attraction is sufficiently strong, and finite groups may keep cohesion in open space as most natural groups do. These models yield a more complex phase diagram where collectively moving groups may assume gas-like, liquid-like or even moving crystal states as the two parameters controlling alignment and cohesion are varied.

So, it remains important to know how individuals make behavioral choices when interacting with others, not only from a social ethology and cognitive viewpoint, but also because i) different behavioral rules may make a difference in small enough groups and ii) the analysis of local-scale data that this requires may lead to discover features eventually found to give rise to different qualitative collective properties. A recent instance can be found in the results on the structure of starling flocks gathered by Ballerini et al. [25]: They have ignited an ongoing debate about the possibility that individuals might interact mostly with neighbors determined by topological rules and not by metric criteria as assumed in most models. While this message has intrinsic value for the study of decision-making processes in animal groups, it was also shown recently that such metric-free, topological interactions are relevant, in the sense that they give rise to collective properties that are qualitatively different from those of metric models [26]. Thus, in this case, an individual-level ingredient suggested by data, which had been only partially and theoretically considered before [6], [7], [27], defines new classes of collective properties. Given that animals are likely to possess more sophisticated behavior than, say, sub-cellular filaments displaced by molecular motors, one can expect more hidden features to play an important role at the collective level. This is a central finding of the recent work by Katz et al. where a careful analysis of groups of two and three fish revealed that the mechanisms at play are, at least in the golden shiners studied there, much more subtly intertwined that in existing fish models [28]. Indeed they concluded that alignment emerges from attraction and repulsion as opposed to being an explicit tendency among fish. Whether fish display some mechanisms of active alignment or only attraction/repulsion is likely to lead to different patterns as interactions accumulate over time. In short, extracting interaction rules from individual scale data is crucial not only for animal behavior studies, but also because heretofore overlooked features can be found decisive in governing the emergent collective properties of moving animal groups.

Here, we assess the power of a bottom-up methodology to build models for animal group motion from data gathered at the individual scale in groups of increasing sizes. We use data obtained by recording the motion of barred flagtails ( *Kuhlia mugil*) in a tank. In natural conditions, the barred flagtail form schools with a few thousands individuals along the reef margin of rocky shorelines, from just below the breaking surf to a depth of a few meters. However the size of these schools is much smaller than in species like the sardine or the Atlantic herring.

Our analysis is incremental: in a previous work we characterized the spontaneous behavior of a single fish, including wall-avoidance behavior [29]. Here, using pairs of fish, we first characterize the response function of one fish depending on the position and orientation of the other fish. Then we calibrate multiple fish interactions, using data in larger groups. At each step, the already-determined factors and parameters are kept unchanged and the new terms introduced in the stimulus-response function and the corresponding new parameters are determined from data with nonlinear regression routines (see Statistical Analysis in Materials and Methods). The resulting model is validated by comparing extensive simulations to the original data. Often, different functional forms are tested and we determine which one is most faithful to the data. When no significant difference is found, the simplest version is retained, following a principle of parsimony.

## Results

### Experimental observations and model basics

**Figure 1:**
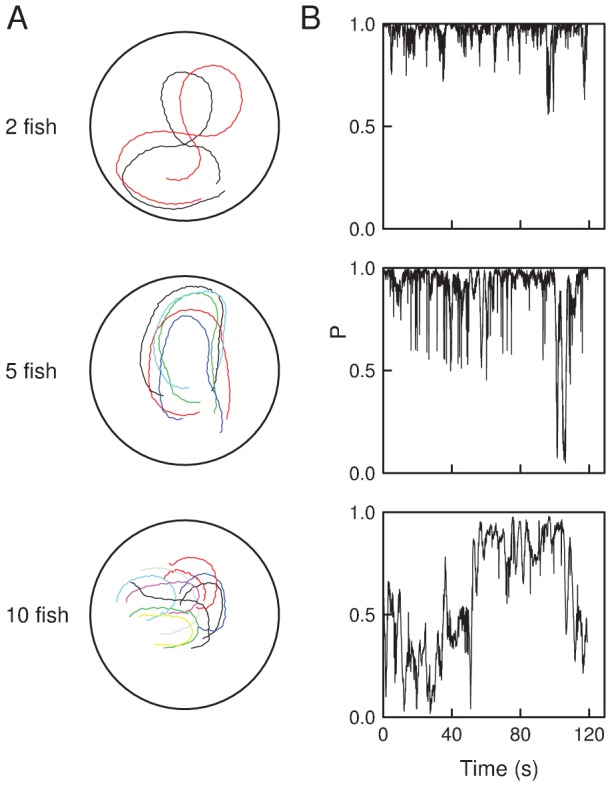
Basic experimental observations. (A) Illustrations of typical fish trajectories in the tank, in groups of 2, 5 and 10 fish, over 9, 5 and 3 seconds respectively. The similarity of trajectories reflect schooling behavior. (B) Time series of the group polarization 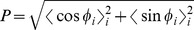, where 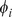 is the heading of fish 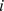. In groups of 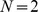, fast swimming fish are nearly perfectly aligned at all times, whereas in larger groups, the alignment is interspersed by desynchronization events.

Experiments with 1 to 30 fish were performed in shallow circular swimming pools that let the fish form quasi 2-dimensional schools (see Fig. 1A and Video S1, S2, S3, S4). At the collective level, we observe a transition from schooling to shoaling behavior when the density of fish increases in the tank: the group polarization 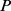, which measures the degree of alignment, is high in groups of two and five fish, even if sometimes we do observe some breaks in the synchronization, while in larger groups, when 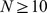, it remains low (Fig. 1B). Within each group size, we notice some variability, the most striking effect being an increase of the synchronization level with the individuals velocity in groups of two fish.

For every group size, fish move continuously and quickly synchronize their speed to a well defined, but replicate-dependent value (Fig. S1). The fish trajectories are smooth, differentiable and the instantaneous speed 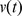 has a well-defined mean 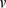 and root mean square fluctuations of about 10–20% which are found to be uncorrelated to 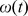, the angular velocity of the fish orientation (Fig. S2). On this basis, fish can be modeled as self-propelled particles moving in 2D space at constant speed 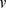 and the only dynamical variable retained is 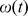. Moreover, since the recorded trajectories, be they extracted from a single fish or from small groups in the tank, are always irregular/stochastic, our model takes the form of coupled stochastic differential equations for the angular velocities of each fish. Note that if noise acts on 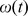 rather than the fish position or heading, trajectories are smooth and differentiable, as observed.

### Single fish behavior and wall avoidance

**Figure 2:**
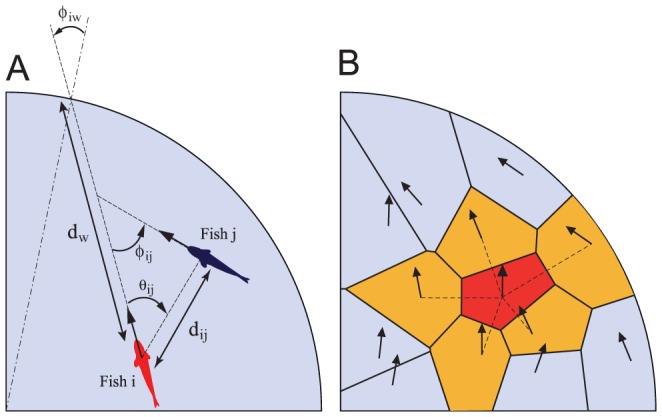
Quantities used in the model formulation. (A) The distance 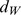 separates the position of the focal fish 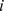 from its current point of impact on the wall; 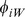 is the angle between the heading of fish 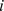 and the angular position of this point of impact with respect to the center of the tank. Neighboring fish 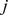 is at distance 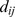 from fish 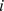; 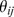 is the angle between the angular position of fish 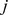 with respect to fish 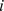 and the heading of fish 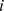. The relative heading of fish 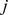 compared to the focal fish 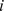 is 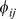. (B) Illustration of a Voronoi neighborhood. Fish headings are indicated by arrows. The focal fish is under the influence of its five neighbors in the Voronoi tessellation (dotted lines), one of which is near the tank wall.

We have shown elsewhere that single fish trajectories in barred flagtails are very well described by an Ornstein-Uhlenbeck process acting on the instantaneous curvature, or, equivalently, on 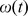 [29]. When the fish is away from the tank wall, the distribution of 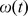 is nearly Gaussian with zero mean and variance 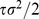, where 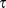 is the characteristic time of the (exponentially decaying) autocorrelation function of 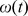. To avoid collisions with the tank walls, we found that a single fish adjusts its current turning speed 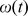 towards a (time-dependent) target value 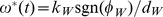 where 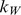 is a parameter, 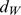 is the distance to the point of impact on the wall should the fish continue moving straight ahead, and 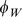 is the angle between the current heading of the fish and the normal to the point of impact (see Fig. 2A). In short, 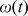 obeys the stochastic differential equation:
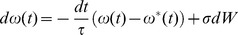
(1)where 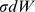 is a Wiener process of variance 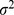 reflecting the stochasticity of the behavioral response. Non-linear regression analysis of the above model against our experimental data yielded excellent agreement and accurate estimations of 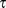 and 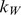. Note that in the present work we adopted a slightly different form for the wall avoidance term with regards to the exponentially decreasing one of Ref. [29], since it actually prevents fish from crossing the tank boundary, while both ansatz are similar as fish moves away from tank walls (Fig. S8A).

### Pair interactions

The stimulus/response function of a single fish in the tank is directly expressed by how 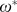 varies with the relative position of the fish and the wall. We now assume that this framework holds when two fish 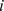 and 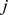 are present in the tank by defining how, for fish 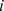, its turning speed 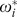 is modulated by the combined stimuli due to the wall and to fish 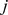. Almost all existing fish behavior models, on the basis of common sense, intuition, and sometimes experimental evidence [30]–[37], offer a combination of three basic ingredients: short distance repulsion (to avoid collisions), alignment for intermediate distances, and attraction up to some maximal range. Here, we dispose of repulsion not only because we want to allow for the rare experimentally observed over- and under-passings events, but mostly because we do not need to incorporate it explicitly to avoid collisions (see below and Video S1, S2, S3, S4). In contrast with most existing “zonal” models, and because there is little cognitive/physiological evidence for a sudden switch between alignment and attraction, we want to allow for continuous, distance-dependent weighting between alignment and attraction in agreement with the recent findings of Katz et al. [28]. These two factors a priori depend on the geometrical quantities defining the location of fish 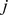 from the viewpoint of fish 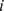: their distance 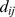 is involved, but also 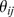, the angular position of fish 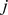 with respect to 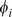, the current heading of fish 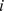, as well as their relative heading difference 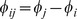 (Fig. 2A). The main angular variable for explicit alignment is, as usual, 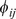, whereas for attraction it is 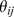; both may also depend on 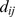. The stimulus/response function 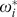 of fish 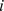 thus combines a priori wall avoidance, alignment and attraction in some unknown function with parameters 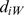 and 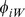 (reaction to the wall), 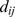, 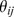 and 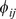: 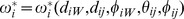.

Next, in the spirit of an expansion around the no-interaction case, we write the expression for 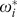 above as the sum of three terms:
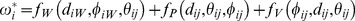
(2)where the “main” variables have been placed first for each term. The wall avoidance term 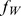 depends explicitly on 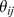 to reflect a possible screening of the wall by the other fish. We have tested the influence of this by introducing a 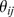 dependence in the wall avoidance term determined for the single-fish behavior. Essentially, 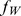 was made smaller for 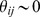. But this brought no significant improvement, so we keep 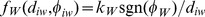 as found previously.

On general grounds, one expects that the relative importance of the positional interaction 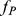 (attraction) to the velocity interaction 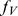 (alignment) increases with 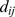. Given that the fish are constrained in a rather small tank, a limited range of inter-distances is effectively explored. In the spirit, again, of a small-distance expansion, a satisfactory choice is given by a linear dependence of 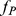 on 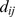, while 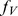 is independent of 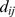. Of course, such a functional choice cannot be correct at large distances since then 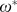 would take large unrealistic values, meaning that the fish would spend enormous amounts of energy turning toward a distant “neighbor” (see the Discussion for more comments on this point).

The attraction interaction 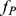 must depend on 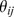, the relative angle with the other fish position: it is reasonable to assume that a fish is not attracted much towards a neighbor located behind, and of course this term must be zero when the other fish is right ahead, yielding 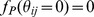. A simple, compatible, trigonometric function representing the leading term of a Fourier expansion is the sine function. We thus write 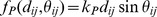 where 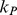 is a parameter controlling the weight of the positional information. Finally, we neglect the possible dependence on 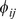: the way a fish would turn toward the position of a neighbor does not depend on the orientation of that fish. This is especially natural when this interaction dominates, i.e. when the neighbor is far away. Moreover knowing the other fish orientation is a cognitively expensive and/or time consuming process at larger distances.

The alignment interaction is mostly characterized by its functional dependence on 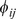. The main constraint here is that 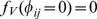 (the two fish are then already aligned). Here again, the simplest choice is 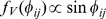 as in most models [8]–[10]. Including higher harmonics (e.g. 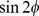) would allow to account for the few observed nematic alignment events where a fish remains anti-aligned with its neighbors. However, incorporating this term did not improve the faithfulness of the model to our dataset, so we keep only the leading sine function. In principle, the strength of alignment can also depend on 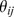: less attention may be paid to “back neighbors”. We have tested simple and reasonable choices for the dependence of 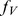 on 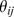, e.g. 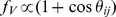, but this did not lead to significant improvement so we kept no angular position dependence in the alignment interaction. We thus write, finally: 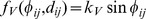 where 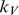 is a parameter controlling the weight of the orientational information.

To summarize the case of two fish 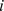 and 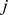, the stimulus/response function 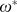 in the general evolution equation (1) is thus finally written:
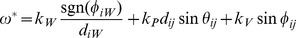
(3)


**Figure 3:**
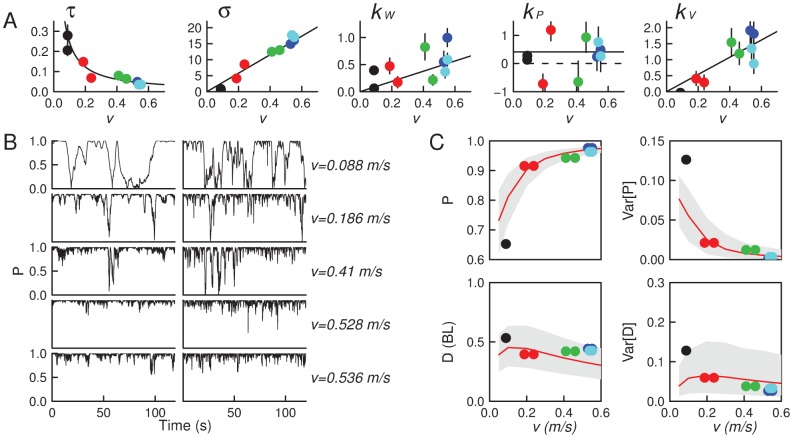
Parameter estimation and validation of the model for groups of 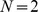 fish. (A) Determination of the parameters of the model defined by Eqs. (1) and (3) from data obtained on pairs of fish (see Statistical Analysis). The values of parameters 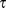, 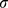, 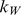, 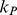 and 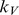 were estimated for each fish separately, and are reported as a function of fish speed (one color per replicate). This reveals the functional dependence of each parameter on the swimming speed 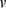. (B) Time series of the alignment between two fish (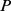) for each experiment (left) and corresponding model simulations (right), ordered by increasing fish speed. Speed is expressed in fish body lengths per second. (C) Comparison between model predictions and experimental data for the time-averaged alignment (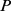) and time-averaged distance (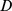) between the two fish as function of swimming speed (color dots : data points, same colors as in A. Red line: predicted mean and gray area : 95% quantiles (see Model Validation)).

Using nonlinear regression analysis, the faithfulness to our data of the model consisting of Eqs. (1) and (3) was found very good for each of our two-fish recordings and the 5 parameters 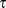, 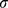, 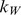, 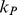 and 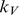 were estimated for each fish. We find clear dependences of the estimated parameters on 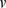, the average speed of each fish (see Fig. 3A). In particular, 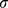, 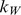, and 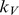 are found proportional to 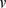, whereas 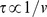 and no significant 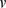-dependence appears for 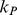. Results regarding this last parameter are the least convincing, with a large dispersion of individual values. This is mostly due to the confinement of fish in the tank: the positional interaction never dominates alignment, preventing its accurate estimation. Nevertheless it is crucial to note here that without these positional interactions the model fails to match the data. Furthermore, we have tested *a posteriori* our ansatz by testing each contribution (either wall avoidance, neighbor position or neighbor orientation) after the other twos have been subtracted from the fish response according to Eq. (3). Results show an excellent agreement between our ansatz and the mean fish response (for more details see Fig. S8 B–D).

Note that these results mean also that the wall avoidance is actually governed by 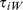, the time it would take the fish 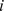 to hit the wall, rather than the distance 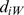. Conversely, 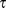, the relaxation time of the angular velocity, is better expressed as the ratio between a characteristic length 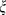 and the speed 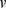. These 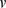-dependences were then incorporated explicitly in the model:
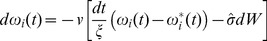
(4)with
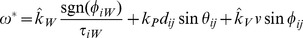
(5)where 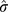, 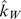 and 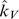 are now constants over all fish. Running again our nonlinear regressions using this form, and using data for all replicate, allows for a more accurate estimation of the parameters 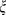, 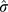, 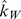, 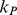 and 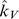 now the same for all fish. We find 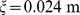, 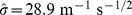, 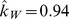, 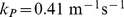 and 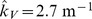.

To validate this experimental finding, these parameter values were used in simulations of the model which were compared directly to the data. Good agreement is found not only for statistical quantifiers of the emergent synchronization between the two fish (see Fig. 3C), but in fact also for the dynamics: see for instance Video S1, S2, S5, S6 and the time series of polarization which show the same intermittent behavior (Fig. 3B). We emphasize that the model captures the experimental observation that the orientational order is lower when the swimming speed is lower, and is better in faster groups (Fig. 3B, C).

### Multiple fish interactions

Can multiple-fish interactions be factorized into pairs? This is often taken for granted, following a typical physics approach where this assumption is routinely made. However, recent work has suggested that this is not valid when describing pedestrian interactions in a crowd [38]. Even more recently, Katz et al. argued that this is also the case for groups of three golden shiners [28] (but see [39] for the case of birds). Here, our data set is too small to allow for an in-depth analysis of group behavior at the level of detail that was accomplished above for two fish, mostly because many more variables are involved, but the quality of the pair approximation can be evaluated a posteriori. Assuming that multiple fish interactions are indeed essentially made of the sum of the pair interactions involved, Eq. (5) is extended to
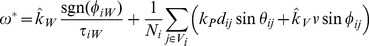
(6)where 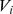 is the (current) neighborhood of fish 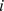 which contains 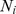 individuals. In our observations with 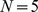 fish, individuals mostly stayed together, suggesting that individuals remains aware of all others. Using all-to-all, equal-weight coupling, we found good agreement between data and simulations of Eqs. (4) and (6) (see Fig. S3). This justifies a posteriori the factorization in pairs and the use of two-fish parameters for 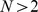 groups, but also the overall normalization factor 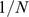 in Eq. (6), which indicates that, in the stimulus response of a fish, wall avoidance and the averaged influence of neighbors keep, on average, the same relative importance irrespective of the group size. The raw, “force-like” un-normalized superposition would yield too strong a coupling.

For the larger group sizes, all-to-all equal-weight coupling quickly becomes unrealistic, and one must determine the set of neighbors a fish interacts with. In principle, abundant data recorded in larger tanks would allow to discriminate between alternative choices, but our experimental recordings are too short for this. Nevertheless, many choices can be eliminated: the usual one, which consists in cutting off interactions at fixed distances (zonal models), is inconsistent with our continuous weighting of alignment and attraction with fish inter-distance. Based on an analysis of starling flocks, Ballerini et al. have argued that these birds actually pay attention to their 6–8 closest neighbors, irrespective of the density of the flock [25]. Coming back to our observations, this non-metric choice of neighbors can, however, lead to unrealistic situations when, for instance, a fish is leading a small group, since then this fish will only pay attention to those behind, even if individuals are located at intermediate distances ahead (but see Fig. S7). A simple, reasonable, non-metric solution is that of neighbors determined by the Voronoi tessellation around each individual: this allows for continuous weighting between alignment and attraction and avoids the caveat mentioned above in the case of a fixed number of closest neighbors. Moreover, given the rather small inter-distances observed, individuals beyond the first shell of Voronoi neighbors are largely screened out, so that our final choice was that of the first shell of Voronoi neighbors (see Fig. 2B). Using this, the validation of the model simulated with 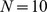 fish using the 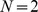 parameters is again quite satisfactory (see Fig. S3).

**Figure 4:**
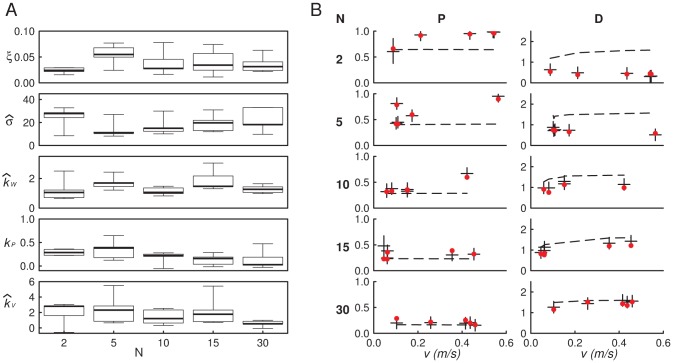
Quantification of group size effect for 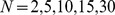 fish. (A) The five parameters 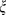, 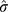, 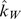, 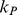 and 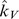 are reported for each replicate, as a function of group size. The first shell of Voronoi neighbors was used. The three parameters reflecting the autonomous part of the behavior (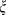 : persistence length, 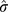: variance of noise and 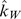: wall avoidance strength) do not show systematic variations with group size. Interaction strength parameters (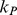: positional interaction, 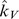: orientational interaction) clearly decrease with group size. (B) Comparisons between model predictions and experimental data using the replicate-based parameters found in A. Left: global polarization 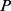, Right: mean inter-individual distance 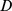 (in meters). (Red circles: data, horizontal bars: predicted means, vertical bars: 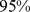 confidence interval, dotted line: predictions under the null model with no interactions). Model predictions were computed by averaging 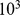 different numerical simulation (with Euler timestepping) for each replicate, starting from the experimental initial conditions, see Model Predictions.

This is however not true anymore for larger groups which display too high a polarization when using the 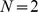 parameters (whereas distance predictions remains satisfactory, see Fig. S3). Our approach actually allows to further investigate this discrepancy. We estimate the parameters at the individual scale for each fish with our nonlinear least-square procedure using the Ito-integrated version of the Ornstein-Uhlenbeck process of Eqs (4) and (6) for each fish time series (see Statistical Analysis). Thanks to this parametric inversion strategy, we have been able to extract the parameter values for each replicate separately (Fig. 4A). The model predictions with these replicate-based parameters yield a near-perfect match with the data (Fig. 4B). The results confirm that, within the limits of statistical accuracy, the parameters and their v-dependence remain about the same up to N = 10, in agreement with the above findings ; but in larger groups there is a decreased tendency of fish to react to their neighbors, which both concerns the alignment and positional interactions (Fig. 4A).

## Discussion

Characterizing and modeling the interactions between individuals and their behavioral consequences is a crucial step to understand the emergence of complex collective animal behaviors. With the recent progress in tracking technologies, high precision datasets on moving animal groups are now available, thus opening the way to a fine-scale analysis of individual behavior [37], [40]–[42]. Here we adopted a bottom-up modeling strategy for deciphering interactions in fish shoaling together. This strategy is based on a step-by-step quantification of the spontaneous motion of a single fish and of the combined effects of local interactions with neighbors and obstacles on individuals motion. At each step, one model ingredient is considered and checked against experimental data. The required parameters are determined using a dedicated inversion procedure and the numerical values of these parameters are kept unchanged in the following steps, yielding, in the end, a model without any free parameter. Such an incremental procedure fosters the explicit enunciation of the rationale behind each functional choice, and differs from searching the best set of free parameters to fit large-group data [10], [43]. Proceeding step by step also puts stronger constraints on matching, since the incorporation of additional behavioral features at each step assumes the stability of the previously explored behaviors and of the corresponding model parameters. Using pairs of fish, we were able to show how positional and directional stimuli combine, and the crucial role of the swimming speed in the alignment interaction. At intermediate sizes, multiple fish interactions could be faithfully factorized into pair interactions albeit in a normalized form. However we found that at even larger group sizes our incremental modeling approach fails to accurately reproduce the collective dynamics.

We explored this point further, still considering the statistical behavior of each fish separately, but only using the data corresponding to the large-group experiments. We concluded that our model could still grasp the observed individual and collective features but with smaller positional and alignment coefficients. We believe that this decrease in reactivity to neighbors is a consequence of the high density already imposed by confinement effects. Indeed, our model predicts that large groups adopting the high neighbor reactivity found in smaller groups would remain polarized also in open space, keeping group cohesion with an average distance to neighbors of about two body lengths (Fig. S6). Since the largest groups we observed in the tank are already characterized by such a typical neighbors distance due to confinement effects, we argue that lower interaction strengths may simply indicate the fish vanishing need to actively react to neighbors position and heading in order to maintain a high density. This could be, for instance, a physiological consequence of the density per se: the physiological and behavioral consequences, for an individual, of living in dense groups, known as group effect, have been described in numerous species from insects to vertebrates [44], [45]. Our results investigation suggests that this sensitivity may be represented in a quite straightforward manner, preserving the model shape of Eqs. (4) and (6) and only modifying the interaction parameters. This conjecture, of course, could only be validated by experiments on large groups conducted in open space or larger tanks. While we believe in a positive answer, namely that without too strong a confinement, individuals would react to the perceived neighbors the same way regardless of the overall group size, we leave this question for future investigations on group effect in fish schools.

Our approach yielded a novel type of fish school model whose main features are its built-in balancing mechanism between positional and orientational information, a topological interaction neighborhood, and explicit dependencies on fish speed. Note that similar features were recently uncovered for another species thanks to a novel data analysis procedure [28]. The smooth transition from a dominant alignment reaction when a neighbor is close to attraction when it is far away is in line with a simple additive physiological integration of both information [46]. The linear dependence of the positional interaction strength on fish inter-distance obviously cannot hold for sparse groups, and will have to be modified by introducing a long-distance saturation when dealing with situations where confinement effects are weaker. Even if we claim that a Voronoi neighborhood was the best choice to account for our data thus extending the relevance of topological interactions, we also checked that our conclusions were robust against this choice, by testing a simple K-Nearest Neighbors network of interactions (which remains topological [25]). We computed the model predictions with the parameters estimated for groups of N = 2 fish, but considering only the K nearest neighbors for increasing values of K (K = 1 to 7, and 10). The results are reported in Fig. S7 ; the main impact of a lower level of connectivity is a decrease of polarization, but it does not lead to better predictions at the collective scale. Interestingly, the best predictions were found with a number of nearest neighbors that corresponds to the average number of neighbors belonging to the first shell in a Voronoi neighborhood (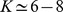, Fig S7–B). This number of influential nearest neighbors is remarkably similar to the one found in starlings [25] and in contrast with recent results found by Herbert-Read et al. in mosquito fish [47]. Further dedicated experiments will be required to discriminate between alternative choices of the relevant neighborhood.

The speed dependence of the parameters, directly derived from our data, is in contrast with most previous fish school models. It leads to an increase of group polarization with swimming speed, a direct consequence of the predominance of alignment at high speed (see Video S7). In natural conditions, this mechanism could be involved in the transitions from shoaling at low speed often associated with feeding behavior to polarized schooling at high speed associated with searching for food. Such speed change could also be elicited by the detection of a threat and abrupt transitions can occur when fish suddenly increase their speed, for instance generating a flash expansion (see Video S8). The question of whether the propagation of such an excitation wave within large schools can generate an efficient collective evasion call for further experimental tests [48].

The reason why our approach was fruitful in spite of the limited amount of data available lies largely in the suitable properties of the behavior of the fish studied: the smooth fluctuations of tangential speed and their de-correlation from angular velocity variations were essential in limiting the number of variables at play but also allowed for a faithful account of single fish behavior by a simple Ornstein-Uhlenbeck process. Clearly it is likely that more complicated solutions will be needed for other species where tangential and angular accelerations are intimately coupled and/or the underlying stochastic process is not as transparent [28]. Nevertheless, we expect that, pending sufficient amounts of data, our approach could be successfully applied to more complex situations occurring in various biological systems at different scales of organization.

## Materials and Methods

### Ethics statement

Our experiments were all carried out in full accordance with the ethical guidelines of our research institutions and comply with the European legislation for animal welfare. The welfare of fishes in the tanks was optimized with a continuous seawater flow, a suitable temperature, and oxygen content. The maximum density in the holding tank was lower than 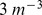. During the experiments, low mortality occurred (five individuals). At the end of the experiment, the fish were released at their capture site.

### Experimental procedures and data collection

The experiments were performed from April to June 2001 at the Sea Turtle Survey and Discovery Centre of Reunion Island. Barred flagtail Kuhlia mugil (Forster) were caught in March 2001 in the coastal area around Reunion Island. 80–100 fishes were conveyed to the marine station and housed in a holding tank of 4 m diameter and 1.2 m depth. Fishes were fed daily ad libitum with a mixture of aquaria flake-food and pieces of fish flesh. Fishes were considered acclimatized when all of them feed on the aquaria flake-food. This weaning period lasted 15 days. Experiments were performed in a circular tank similar to the holding tank. Opaque curtains were placed around and above the tank to obtain diffuse lighting and to reduce external disturbances from the environment. The tank was supplied with a continuous flow of seawater [49]. Since currents may influence fish behavior, the seawater inlet pipe was placed vertically and the water flow was stopped throughout the observation periods. A digital video camera (Sony model CDR-TRV 900E) was fixed at 5 meters above the tank and tilted at 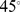 to observe the whole tank. The remotely operated video camera was fitted with a polarizing filter and a wide-angle lens. Groups of N = 1 to 30 fish were introduced in the experimental tank and acclimatized to their new environment for a period of 20 min. Their behavior was then recorded at 24 fps for 2 mins. Prior to each trial, the fish were deprived of food for 12 hours to standardize the hunger level and were transferred to the experimental tank. The relative shallowness of the water ensured quasi two-dimensional motion. Five replicates per group size using different individuals were performed. Eighty per cent of the trials were performed in the morning to avoid possible conditions of strong wind that may disturb the fish, and sunshine that may render light inside the tank unsuitable for video recording. A first data processing consisted in sampling 12 images per second out of the 24 images recorded by the video camera. A custom-made tracking software was then used to extract high-quality, smooth trajectories from the video recordings, with crossing ambiguities resolved by eyes (see Video S3, S4). In order to get even higher precision data, the head position and the orientation of each fish in groups of N = 2 were acquired with a manual tracking software (Video S1, S2).

### Statistical analysis

Model parameters were estimated from each fish time series separately (typical series are shown on Fig. S4). In order to perform the estimation of the parameters 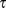, 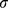, 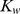, 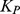 and 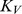 in the stochastic differential equation (1), (3) and (5), we considered its discrete-time version using Ito integration over 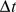, assuming 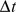 is small enough so that 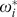 is constant [50]:
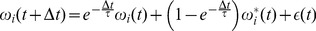
(7)where i = 1,2 and 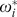 is given by Eq. (3) or (5). Estimates for the parameters were obtained using a standard non-linear least squares procedure (we employed the nls package of the statistical environment R [51]) either separately for each fish using Eq. (3) or for all fish together using Eq. (5). Residuals given by 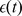 were checked to be Gaussian-distributed (see Fig. S5) and their variance yielded 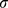.

### Model predictions

The model was simulated within a virtual tank, using the estimates of behavioral parameters extracted by statistical analysis from 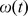 time-series in groups of 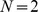 fish. The fish heading (direction of motion) 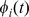 and position 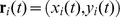 were updated by Euler integration, following:
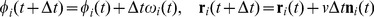
(8)where 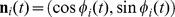.

For each 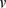 value, 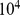 numerical simulations were performed over 120 seconds (a time corresponding to the duration of individual experiments with real fish) with a time step 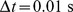. A transient time of 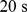 was discarded before measuring statistical averages. We computed the mean value and the variance over time of the global polarization
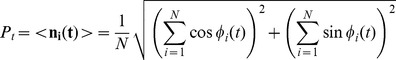
(9)and of the neighbor inter-distance
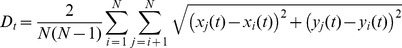
(10)


This yielded an estimation of the expected measures distribution under model hypothesis and over the typical observation time of experiments. We then computed the mean and 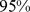 confidence interval of such distributions, to obtain the expected mean and variance (with their confidence intervals) of alignment and of neighbor inter-distance. This provided the check of the model against experimental data. The above procedure was repeated varying the mean speed 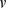 over the range covered by the experimental data, with the results plotted in Fig. 3C. The same procedure was adopted to make predictions for higher group sizes, using the stimulus/response function 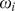 as determined by equation (5) with interacting neighbors defined by first neighbors in a Voronoi tessellation (For a set 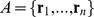 of 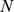 points, Voronoi tessellation divides the space in 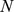 different cells, each the locus of space closer to its center 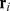 than to any other points in 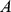: at each time step space is divided in 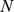 Voronoi cells centered around the 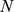 fish position, with Voronoi neighbors being the fish lying in neighboring cells (Fig. 2B). For each experimental replicate, the same measures were repeated with the parameters extracted from the replicate, and the corresponding initial conditions (Fig. 4B).

### Model validation

By construction, our method does not “learn the parameters to make the model fit”, contrasting with a more usual procedure which consists in stating an a priori model and searching a best set of free parameters that optimizes its collective patterns towards the observed collective properties (namely, make the model fit at the collective scale). In such cases, it is known that several models can adjust the data at the collective scale (because the search for best match is unconstrained and can be performed for each model, so that the collective level underdetermines the individual level).

In the present study, once the model has been formulated, that is, once we identified in the experiments with pairs of fish the nature of stimuli (the orientation and relative position of neighboring fish, and how they combine to determine the response of a focal fish), we estimated the values of 5 parameters at the individual scale. So for each fish, we measured its behavioral response (i.e. the change of its turning speed) for each configuration of stimuli encountered in its path.

Only then, we tested whether these parameters measured at the individual level can explain the observations at the collective scale *with no free parameters*. For each group independently, we thus checked that the model allows a quantitative matching concurrently at individual and collective scales. This confirmed that our model calibrated with the parameters estimated from the third derivative of the fish position (i.e. the change in the turning speed) was able to reproduce quantitatively the statistics resulting from the time integration of the coupling between fish (polarization, inter-distance). Moreover the same procedure applied separately on each group size revealed, on the one hand, the dependences of the estimated parameters on the swimming speed (using groups of N = 2 fish), and on the other hand, the modulation of interactions' strength with group size (in the largest groups).

## Supporting Information

Figure S1 Distance travelled by fish as a function of time in 3 different experiments with N = 2 fish (left panel), one N = 5 and one N = 10 experiment (middle and right panel). In any given experiment, fish synchronize their speed, but this value is replicate-dependent.
(EPS)

Figure S2Swimming speed and angular velocity of one fish. Left: Time series of instantaneous speed 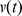 and angular velocity 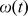 of one fish during a typical experiment (here 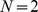), together with the respective histograms. Right: parametric plot of 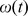 vs. 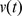. The speed fluctuates relatively mildly around its mean, while 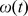 varies wildly. The parametric plot reveals no correlation between the two quantities.(EPS)

Figure S3Comparisons between experimental data at all group sizes and predictions of the model, using the 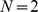 parameters and the first shell of Voronoi neighbors. (Red circles: data, horizontal bars: predicted means, vertical bars: 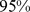 confidence interval, dotted line: predictions under the null model with no interactions, except interactions with wall). For each experimental replicate, 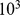 numerical simulations were performed over 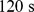 with a time step of 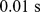. Over each period of 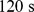 (corresponding to the duration of individual real fish experiments), the mean value of the global polarization and fish inter-distance were calculated, and averaged in time. Very good agreement with experiments is found for 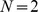 and 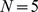. From 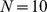 to 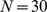, the distance predictions remain correct, but the discrepancy between model predictions and data for the polarization increases with group size. The model predicts too high polarization values especially at large speed values.(EPS)

Figure S4 Example of experimental time-series used to estimate the N = 2 parameters. From top to bottom: turning speed response, wall effect stimulus, positional stimulus and directional stimulus. This shows that the tracking yielded a very good signal to noise ratio.
(EPS)

Figure S5Distribution of residuals for the N = 2 parameters estimation. For each fish in the groups with 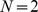, the residuals were plotted in a quantile-quantile plot (normalized experimental quantiles vs theoretical quantiles under the normal hypothesis). The linearity of the plots is a strong indication in favor of a Gaussian distribution of the residuals (with an outlier for fish 2 in experiment 2: M2-2, which exhibits large deviations). This justifies a posteriori the use of a simple Wiener term in the stochastic differential equation describing the model. The estimated variance of the residuals yields an estimate of 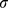.(EPS)

Figure S6Model predictions in open space, using the N = 2 parameters for every group size and the first shell of Voronoi neighbors. (horizontal bars: predicted medians, vertical bars: 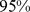 confidence interval, Red circles: experimental data in the tank, dotted line: predictions under the null model in the tank with no interactions, except interactions with wall). (A) : global polarization P, (B) : average inter-individual distance D (in meters), (C) : average distance to the nearest neighbors D(NN) (in meters). As swimming speed increases a high level of polarization with about the same nearest neighbor distance is observed for all group sizes. These results strongly contrasts with the experimental observations suggesting a decrease in reactivity to neighbors as a consequence of the high density already imposed by confinement effects.(EPS)

Figure S7Tests of the alternative neighborhood definition, based on K-Nearest neighbors with 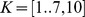. We computed the prediction errors for polarization and distances cumulated over all groups and sizes, namely the sum of square differences between the observed values and the predicted values, as those shown in Fig. 4B. The prediction errors for distances are reported in blue, and the prediction errors for polarization are reported in black. The prediction errors for the Voronoi definition of influential neighbors are also reported, for reference (dotted lines). (A) First, to check whether the loss of polarization in large groups can be explained by restricting the neighborhood to the few first nearest neighbors as found by Herbert-Read et al. [47], we computed the predictions of the model using the N = 2 parameters, with 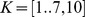. Indeed, if fish were to react strongly but only to the 3 nearest neighbors, the prediction error for the distances can be about as low as for the Voronoi neighbors. However, this is not the case for the polarization error, which remains by far greater than with replicate-dependent parameters. Actually, interactions with fewer neighbors can impede the global polarization, but still allows for local polarization between nearest neighbors, a picture which does not correspond to the homogeneous loss of polarization noticeable in movie S4. We conclude that the lower polarization in large groups cannot be simply explained by considering a weaker coupling due to a limited number of influential neighbors. (B) As a complementary check, we also performed the complete inversion procedure over all groups, and for each value of 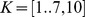, deriving in each case the model predictions (as for Fig. 4, using here 100 simulated series for each of the 25 groups and for each of the eight values of K). Doing this, we observe that the prediction errors reach minimal values for about 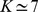, and are then of same order as the prediction errors under the Voronoi neighborhood hypothesis. We note that the Voronoi definition yields a number of neighbors which fluctuates with time around this value, and that the fish are more or less homogeneously distributed in the tank. We conclude that the two definitions of neighborhood practically overlap in the present experimental setup.(EPS)

Figure S8Validation of the ansatzes. (A) Strength of the wall avoidance term 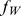 in the absence of strong positional and directional stimuli from the neighboring fish as a function of wall distance 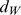. Data (black circles) have been extracted considering one fish in the fastest 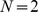 group under the condition 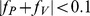, so that 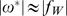. We estimated the response 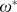 from the turning speed by making use of Eq. (7). The two fitting lines represent the best fit for the ansatz adopted in this paper (black, 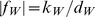, 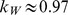) and for the one of Ref. [29] (blue, 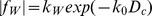, 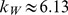 and 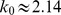). While the sharp decrease of 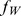 with 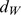 is obvious, the scarcity of our data and the stochastic nature of the effective fish response do not allow to detect the fine difference between the two ansatzes, which yield about the same average reaction. (B, C, D) Residual fish responses to tank boundaries, neighbor position and neighbor orientation for all 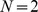 groups (for the sake of clarity, we have confined our analysis to couples of fish to avoid any ambiguity on neighboring relations). For each fish 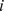 at each time 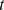, the fish response 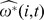, and the three stimuli 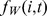, 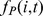 and 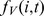 were estimated from the data by making use of Eq. (5) and (7), and using the estimated parameters reported in Fig. 3A. (B) Wall response 
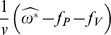
 as a function of the wall stimulus 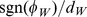. (C) Positional response 
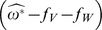
 as a function of the positional stimulus 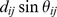. (D) Directional response 
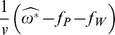
 as a function of the directional stimulus 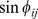. The 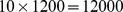 data points have been averaged over discrete bins (full dots: mean, vertical bars: standard deviation) to highlight the expected linear relation between residual responses and stimuli. Red lines correspond to the results of the regression analysis using the full model (see main text), showing a conclusive agreement between our chosen ansatzes and the mean fish response.(EPS)

Video S1Video recording of an experiment with N = 2 fish swimming at low speed (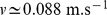). Light blue lines correspond to the fine-grained hand-tracking. Interactions among fish lead to a succession of attraction and alignment phases.(MOV)

Video S2Video recording of an experiment with N = 2 fish swimming at a higher speed (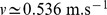). Fish exhibit a high level of synchronization. The associated time series are shown in Fig. 1B (polarization).(MOV)

Video S3Video recording of an experiment with N = 5 fish swimming at high speed (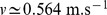). The white dots correspond to the position of the heads of the fish detected by the tracking software. Fish maintain a high level of synchronization and as a consequence group polarization is high.(MOV)

Video S4Video recording of an experiment with N = 15 fish swimming at high speed (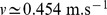). The white dots correspond to the position of the heads of the fish detected by the tracking software. Fish do not fully synchronize their motion and as a consequence group polarization is lower than in smaller groups.(MOV)

Video S5Simulation of interactions in a group of N = 2 fish swimming at low speed (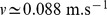). The parameters correspond to those in Figure S1. The polarization of fish motion is low with a succession of attraction and alignment phases.(MOV)

Video S6Simulation of interactions in a group of N = 2 fish swimming at higher speed (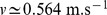). The parameters correspond to those in Figure S2. The polarization of fish motion is high.(MOV)

Video S7Transition in group polarization induced by velocity change. The simulation was performed in unbounded conditions with a group of 100 fish with parameters 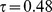, 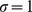, 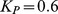, 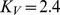. The swimming speed of all fish is initially set to 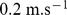, linearly increases to 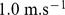 in 60 s, is maintained to this value for 30 s, and then decreases back to 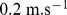 in 60 s. Movie time is 5× real time. The group switches from shoaling to schooling dynamics (and back) as a consequence of the increase (and decrease) of the swimming speed. As it increases, the relative weight of the orientational interaction dominates the positional interaction, leading to a better polarization which triggers a motion of the center of mass of the group.(MOV)

Video S8Transition in group polarization induced by a sudden velocity increase. The simulation was performed in the same conditions and with the same parameters as those used in Video S7, but with a different time profile of the change of the swimming speed (from 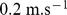 initially, the speed abruptly increases to 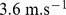 in 0.2 s, then slowly decreases back to 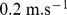 in 15 s). When the swimming speed suddenly increases over a short time interval in a shoaling group, the alignment interaction becomes abruptly dominant over position interaction, and neighboring fish align to each other. This polarization remains local due to the lack of time to build up over the entire group so that the initial isotropic distribution of headings is conserved for a short time, and a flash-expansion pattern arises. After the speed has decreased, the group returns to shoaling. Video S7 and S8 show that the speed-dependencies can trigger very different collective responses, depending on the rate of change. This control of collective behavioral response by speed is a parsimonious, effective, and robust mechanism. It also suggests further experiments aimed at identifying which external factors can affect individual speed (light, food presence or depletion, predators strike, …), and at elucidating the propagation of speed changes to the neighbors.(MOV)
